# Neurobiological characteristics of treatment resistance and predictors of treatment tolerability for schizophrenia

**DOI:** 10.1192/j.eurpsy.2025.107

**Published:** 2025-08-26

**Authors:** E. Van Assche

**Affiliations:** Department of Psychiatry, University of Münster, Münster, Germany

## Abstract

**Abstract:**

Schizophrenia is a chronic disorder with a massive impact on quality of life. The prevalence of treatment resistance has been estimated around 30%. Treatment resistance is typically defined as non-response to 2 antipsychotics *of adequate dose and duration.* However, clinical reality often shows that the definition at an individual level is more complex and a personalised approach is needed.

We present a literature review discussing clinical, neurobiological and pharmacogenetic aspects relevant to treatment resistance.

Our overview highlights the need for indiviudalised assessement of patient needs. We discuss pharmacogenetic aspects related to treatment respons and tolerability, including Clozapine. We focus on indicators for stratification and treatment optimization in the context of personalised medicine. In addition, we present evidence for other augmenting treatment modalities in the context of treatment resistance for schizophrenia, such as neuromodulation (e.g., rTMS for acoustic hallucinations).

This talk focuses on the challenges of treatment resistance in schizophrenia and discuesses new insights in the existing treatments and pharmacogenetics, as well as opportunities for treatment augmentation that arise from emerging technologies.

**Disclosure of Interest:**

None Declared

